# Cu (II)-Catalyzed Asymmetric Henry Reaction with a Novel C_1_-Symmetric Aminopinane-Derived Ligand

**DOI:** 10.3390/molecules20046224

**Published:** 2015-04-09

**Authors:** Liudmila Filippova, Yngve Stenstrøm, Trond Vidar Hansen

**Affiliations:** 1 Department of Chemistry, Biology and Food Science, Norwegian University of Life Sciences, P.O. Box 5003, N-1432 Ås, Norway; E-Mail: liudmila.filippova@nmbu.no; 2 Department of Pharmaceutical Chemistry, School of Pharmacy, University of Oslo, P.O. Box 1068, Blindern, N-0316 Oslo, Norway; E-Mail: t.v.hansen@farmasi.uio.no

**Keywords:** asymmetric catalysis, Henry reaction, nitrooaldol, chiral ligand, copper

## Abstract

A novel C_1_-symmetric dinitrogen ligand was synthesized in high yield from commercially available (1*R*,2*R*,3*R*,5*S*)-(−)-isopinocampheylamine and 1-methyl-2-imidazolecarboxaldehyde. In combination with Cu(OAc)_2_∙H_2_O, this new ligand promote the reaction between nitromethane and aliphatic aldehydes with high yields (up to 97%) and moderate enantioselectivities (up to 67% ee). The reactions with benzaldehyde required prolonged reaction time that resulted in diminished yields, but accompanied with ee-values in the 55%–76% range.

## 1. Introduction

The asymmetric Henry (nitroaldol) reaction provides a straightforward entry to enantiomerically enriched β-nitro alcohols, which are valuable intermediates in the synthesis of natural compounds and biologically interesting molecules [1,2]. In particular, the nitroaldol products can be reduced into vicinal amino-alcohols which is a common structural motif found in many pharmaceuticals, such as (−)-pindolol [3], (−)-arbutamine [4], ritonavir [5], (*R*)-salmeterol [6], and epinephrine [7]. In addition, the β-amino alcohol functionality is present in long-chain lipids such as sphingosines [8,9]. Thus, the development of an efficient asymmetric protocol for this type of reaction is of current interest [10,11].

**Figure 1:**
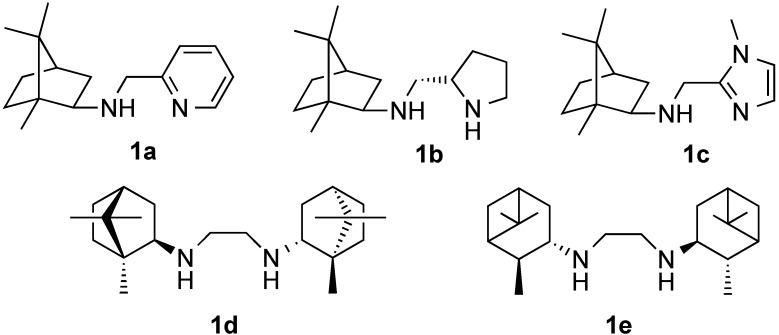
Some camphor and pinane-derived *N*,*N*-ligands.

The first asymmetric procedure for nitroaldol reaction employing a bimetallic Li-La BINOL complex was reported in 1992 by the Shibasaki group [3]. Since then, extensive collection of organocatalysts [12,13,14,15] and transition metal based catalytic systems, *i.e.*, Zn [4,16], Co [17,18] Cr [19,20], Cu [21,22,23,24,25,26,27,28,29,30,31,32,33,34,35,36,37,38,39,40,41,42,43,44,45,46,47,48,49,50], have been developed with variable success. The majority of these metal-based catalysts reported involved the use of copper complexes with either a bi- or polydentate aza-containing chiral ligand (such as BOX-type [21,22,23], diamines [7,24,25,26,27,28,29,30,31,32], Schiff bases [17,33,34,35] amino-alcohols [9,36,37,38,39], amino/iminopyridines [40,41,42,43,44], sulfonamides [45,46]). These systems have gained some applications, but some limitations still exist. For example, multistep synthetic procedures are often necessary for the preparation of some of the ligands, and obtaining the catalyst in both enantiomeric forms is sometimes a challenge. Moreover, many of the catalytic systems display poor enantioselectivity together with low yields of the products when employing aliphatic aldehydes as substrates. Therefore, the development of easily obtainable, novel ligands, in both enantiomeric forms, is still desired for this class of aldehydes. In this view, the chiral camphor and pinane based terpenes are examples of convenient building blocks for the development of effective chiral auxiliaries. For instance, the copper complexes with camphor-based dinitrogen ligands **1a** [40], **1b** [7], **1c** [47] and **1d** [48] have been reported to give high level (up to 98%) of asymmetric induction when reacting nitromethane with a broad range of aldehydes, including aliphatic ones (Figure 1). 

**Scheme 1:**
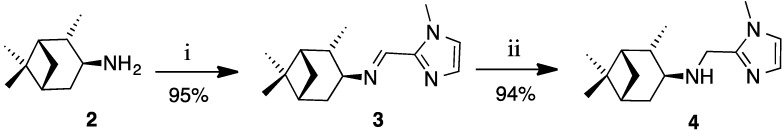
Synthesis of the ligand **4**

Compounds bearing a pinane core have been utilized in some asymmetric transformations such as the enantioselective addition of diethylzinc to aldehydes [51,52,53], aldol condensation [54] and reduction of ketones [55]. Recently, the copper complex of C_2_-symmetric ligand **1e** was reported to promote the asymmetric Henry reaction between nitromethane and aliphatic aldehydes affording nitroaldol products with enantiomeric excess from 57% to 93% ee [56]. Based on the synthesis of **1c** [47], we designed the novel pinane-derived C_1_-symmetric ligand **4** (Scheme 1). Herein, we describe its application in a copper-catalyzed asymmetric nitroaldol reaction. 

## 2. Results and Discussion

The C_1_-symmetric ligand **4** was easily prepared from commercially available (1*R*,2*R*,3*R*,5*S*)-(−)-isopinocampheylamine (**2**) and 1-methyl-2-imidazolecarboxalehyde in a one-pot reaction as outlined in Scheme 1. 

*Reagents and conditions*: (i) 1-methyl-2-imidazolecarboxaldehyde (1 equiv.), MeOH, rt; (ii) NaBH_4_ (3 equiv), MeOH, 0 °C to rt.

Initially, in connection with our interest in the synthesis of aliphatic nitroalcohols, we examined the ability of **4** to promote the nitroaldol reaction between valeraldehyde (**5a**) and nitromethane in the presence of CuCl_2_∙2H_2_O (5 mol %) at 0 °С with DIPEA (1 equiv.) as base additive. The results are summarized in Table 1. When THF was used as solvent, the reaction proceeded smoothly resulting in high yield (92%), but moderate enantioselectivity was observed (Table 1, entry 1). Then Cu(OAc)_2_∙H_2_O was tested to see if there were any influence of the counterions on the reaction. Both copper salts gave similar results in terms of enantioselectivity, but the yields varied significantly depending on the solvent (Table 1, entries 1–4). Most likely, this is due to differences in the solubility of the formed complexes. Hence, the use of other solvents was investigated. Of the solvents tested, *i-*PrOH was the best choice (Table 1, entry 6, 97% yield, 55% ee). Lowering the temperature of the reaction to −20 °С resulted in a significant decrease of the reaction rate with no improvements in enantioselectivity (Table 1, entries 8 and 9). The absolute configuration in the product **6a** was determined based on comparison of the specific optical rotation value with literature [24,25,26,27,28,29,30,31,32].

**Table 1:** Selected experimental conditions for asymmetric Henry reaction between pentanal and nitromethane. 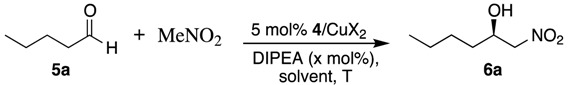

Entry ^a^	Copper Salt	Solvent	T (°C)	Time (h)	DIPEA (mol %)	Yield (%) ^b^	e.e. (%) ^c^
1	CuCl_2_∙2H_2_O	THF	4	20	100	90	53
2	Cu(OAc)_2_∙H_2_O	THF	4	20	100	52	52
3	CuCl_2_∙2H_2_O	EtOH	4	20	100	88	33
4	Cu(OAc)_2_∙H_2_O	EtOH	4	20	100	92	37
5	Cu(OAc)_2_∙H_2_O	CH_2_Cl_2_	4	20	100	68	47
6	Cu(OAc)_2_∙H_2_O	*i*-PrOH	4	20	100	97	55
7	Cu(OAc)_2_∙H_2_O	Et_2_O	4	20	100	63	46
8	Cu(OAc)_2_∙H_2_O	THF	−25	20	100	<5	n.d. ^d^
9	Cu(OAc)_2_∙H_2_O	*i*-PrOH	−25	72	100	88	57
10	Cu(OAc)_2_∙H_2_O	*i*-PrOH	4	2	100	68	57
11	Cu(OAc)_2_∙H_2_O	*i*-PrOH	4	4	100	88	56
12	Cu(OAc)_2_∙H_2_O	*i*-PrOH	4	6	100	85	55
13	Cu(OAc)_2_∙H_2_O	*i*-PrOH	4	8	100	89	57
15	Cu(OAc)_2_∙H_2_O	*i*-PrOH	4	20	0	36	56
16	Cu(OAc)_2_∙H_2_O	*i*-PrOH	4	20	5	88	57
17	Cu(OAc)_2_∙H_2_O	*i*-PrOH	4	20	10	98	56

^a^ The reactions were carried out on 0.5 mmol scale of valeraldehyde with 10 equiv. of nitromethane in 2 mL of solvent. ^b^ Isolated product. ^c^ Enantiomeric excesses by HPLC analyis using a column with a chiral stationary phase. ^d^ Not determined.

The Henry reaction is reversible [1]. The enantiomeric excess of the nitroaldol adducts can decrease as a result of the retro-nitroaldol reaction. Hence, a time-course study was performed to investigate the possibility of reversibility. The reaction was almost complete within four hours, while the ee-values of the product **6a** remained constant in the conditions explored (Table 1, entry 10–13), indicating the absence of reversibility.

An external base is usually added to promote the reaction. Considering the still low ee of the product observed, and the fact that the excess of additional base can enhance the non-enantioselective reaction pathway [26], the effect of base loading was examined. The addition of even small amounts of external base noticeably improved the reaction rate, while the ee-values of the products were unchanged with increasing amounts of base added. These experiments verified that inhibition of an unselective pathway occurred (Table 1, entries 15–17). The loading of 10 mol % of external base was sufficient to promote the reaction until completion.

Based on the conditions investigated, different aliphatic aldehydes were tested. As shown in Table 2, all of the aldehydes smoothly converted into the nitroaldol products with good to excellent yields. Moderate ee-values ranging from 49% to 67% were observed; the highest ee-value was observed with 2,2-dimethylpropanal (**5c**) (Table 2, entry 3, 67% ee). Again, the absolute configuration in the products **6a–6h** was assigned based on specific optical rotation values.

Then we tested the catalyst system with benzaldehyde (**5h**) (Table 2, entries 8–10). The reaction between **5h** and nitromethane in the presence of ligand **4** and CuCl_2_∙2H_2_O did not reach completion even after 20 hours at 4 °С (Table 2, entry 8). Lowering the temperature of the reaction improved the level of enantioinduction to 76% ee, but drastically diminished the yields to impractical values (Table 2, entries 9–10). Hence, other aromatic aldehydes were not investigated.

Finally, in view to explore the nitroalkane substrate scope, nitroethane was reacted with aliphatic aldehydes employing optimized catalytic procedure (Table 3). The corresponding nitroaldol adducts bearing two stereogenic centers were obtained in high yields (up to 94%) albeit moderate diastereoselectivities favoring *syn*-product and enantioselectivities were observed. Diastereoselectivity was improved when α-branched aldehyde was used (Table 3, entry 2).

**Table 2:** Enantioselective Henry reaction of various aldehydes with nitromethane. 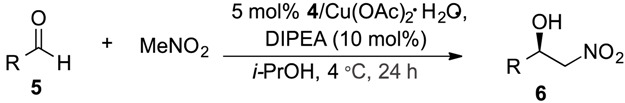

Entry ^a^	R	Aldehyde	Product	Yield (%) ^b^	e.e. (%) ^c^
1	*n*-Bu	5a	6a	91	57
2	*i*-Bu	5b	6b	89	49
3	*t*-Bu	5c	6c	88	67
4	*i-*Pr	5d	6d	83	60
5	*n*-nonyl	5e	6e	75	55
6	*n-*undecyl	5f	6f	82	53
7	*trans*-2-decenyl	5g	6g	65	47
8 ^d^	phenyl	5h	6h	80	38
9 ^e^	phenyl	5h	6h	63	45
10 ^f^	phenyl	5h	6h	10	76

^a^ The reactions were performed on 0.5 mmol scale. ^b^ Isolated yield. ^c^ Determined by HPLC using a column with a chiral stationary phase. ^d^ CuCl_2_∙2H_2_O was used as a copper source. ^e^ The reaction was carried out at −20 °С for 48 h in the presence of 1 equiv. of DIPEA. ^f^ The reaction was carried out at −40 °С for 48 h in the presence of 1 equiv. of DIPEA.

**Table 3:** Diastereoselective Henry reaction using nitroethane. 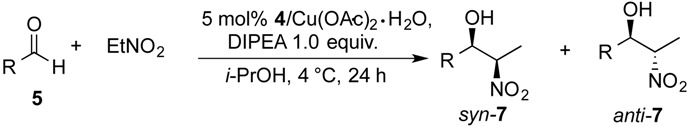

Entry ^a^	R	Product	Yield (%) ^b^	*syn*/*anti* (%) ^c^	ee (%) ^d^
1	*i*-Bu	7a	93	58/42	54/38
2	*i*-Pr	7b	94	81:19	66/34

^a^ The reactions were performed on 0.5 mmol scale. ^b^ Isolated yield. ^c^ Determined by ^1^H-NMR spectroscopy analysis of isolated compound. ^d^ Determined by HPLC analysis using a column with a chiral stationary phase.

## 3. Experimental Section 

All starting reagents and solvents were obtained from Sigma Aldrich and used as purchased without further purification. Analytical TLC was performed using silica gel 60 F_254_ plates (Merck). Flash column chromatography was performed on silica gel 60 (40–63 nm). NMR spectra were recorded on Varian Gemini and Bruker Ascend^TM^ 400 spectrometers 300 MHz and 400 MHz for ^1^H-NMR and 75 MHz and 100 MHz for ^13^C-NMR, respectively. Chemical shifts are reported in ppm downfield from tetramethylsilane relative to CDCl_3_ as internal standard (7.24 ppm for ^1^H and 77.00 for ^13^C). IR spectra (4000–600 cm^−1^) were recorded on a Perkin-Elmer Spectrum BX series FT-IR spectrophotometer using a reflectance cell (HATR). Optical rotations were measured using a 1 mL cell with 1.0 dm path length on Perkin Elmer 341 polarimeter in dedicated solvent. HPLC analyzes were performed on Agilent 1200 Series instrument using chiral OD-H or AD-H columns.

### 3.1. Preparation of Ligands

#### 3.1.1. Synthesis of (*E*)-1-(1-Methyl-1*H*-imidazol-2-yl)-*N*-((1*S*,2*S*,3*S*,5*R*)-2,6,6-trimethylbicyclo[3.1.1]heptan-3-yl)methanimine (**3**)

To (1*R*,2*R*,3*R*,5*S*)-(−)-isopinocampheylamine (**2**) (612 mg, 4.0 mmol) was added of 1-methyl-2-imidazolecarboxaldehyde (440 mg, 4.0 mmol) in 20 ml of dry methanol. The reaction mixture was left stirring at room temperature until no more starting material was detected (controlled by TLC, visualized by ninhydrine stain). Then mixture was directly concentrated under reduced pressure and purified by column chromatography eluting with hexane and ethyl acetate to obtain 930 mg of compound 3 as colourless oil (95% yield). ${\left[\alpha \right]}_{D}^{20}$ = −45.1 (c = 2.0, EtOH). ^1^H-NMR (CDCl_3_, 400MHz, TMS): δ = 8.16 (s, 1H), 7.07 (s, 1H), 6.88 (s, 1H), 3.99 (s, 3H), 3.36–3.33 (m, 1H), 2.38–2.25 (m, 2H), 2.10–2.01 (m, 1H), 1.98–1.91 (m, 1H), 1.89–1.81 (m, 2H), 1.22 (s, 3H), 1.47 (d, *J* = 9.3 Hz, 1H), 1.03 (s, 3H), 0.98 (d, *J* = 8.7 Hz, 3H) ppm. ^13^C-NMR (75 MHz, CDCl_3_, TMS): δ = 149.7, 143.2, 128.9, 124.4, 70.5, 47.5, 44.1, 41.6, 38.7, 36.2, 35.4, 33.6, 27.9, 23.5, 19.8 ppm. 

#### 3.1.2. Synthesis of (1*S*,2*S*,3*S*,5*R*)-2,6,6-Trimethyl-*N*-((1-methyl-1*H*-imidazol-2-yl)methyl)-bicyclo[3.1.1]heptan-3-amine (**4**)

Sodium borohydride (456 mg, 12.1 mmol, 3.2 equiv.) was added in portions to a chilled 0 °C solution of **2** (930 mg, 3.8 mmol) in methanol. The mixture then was allowed to warm to room temperature and left stirred until no more starting material was detected (controlled by TLC, UV detection). The reaction was quenched with aqueous HCl, and MeOH solvent was removed under reduced pressure. The aqueous part was basified until pH = 9–10 and extracted by ethyl acetate, dried over anhydrous Na_2_SO_4_ and evaporated. The crude product was purified by flash column chromatography (hexane: EtOAc 7:3) to obtain 884 mg (94% yield) of **4** as a solid compound. ${\left[\alpha \right]}_{D}^{20}$ = −66.9 (c = 1.85, EtOH). ^1^H-NMR (CDCl_3_, 400 MHz, TMS): δ = 6.88 (s, 1H), 6.78 (s, 1H), 3.80 (dd, *J_1_* = 13.2 Hz, *J_2_* = 24.5 Hz, 2H), 3.69 (s, 3H), 2.88–2.81 (m, 1H), 2.41–2.24 (m, 2H), 1.93–1.90 (m, H), 1.78–1.74 (m, H), 1.64–1.57 (m, H), 1.18 (s, 3H), 1.06 (d, *J* = 7.5 Hz, 3H), 0.93 (s, 3H) ppm. ^13^C-NMR (75 MHz, CDCl_3_, TMS): δ = 147.5, 127.7, 121.7, 57.6, 48.4, 45.7, 44.9, 42.3, 39.1, 37.1, 34.3, 33.3, 28.4, 24.0, 22.1 ppm. HRMS (TOF ES^+^): exact mass calculated for C_15_H_26_N_3_ [M + H]^+^ 248.2126; found 248.2124.

### 3.2. General Procedure for the Enantioselective Henry Reaction

Ligand **4** (6.2 mg, 5 mol %) and Cu(OAc)_2_∙H_2_O (5 mg, 5 mol %) were added to the test tube containing 2 mL of *i*-PrOH and stirred for an hour to obtain a blue solution. Then the test tubes were transferred to a bath at the given reaction temperature and 0.5 mmol of aldehyde, 10 equivalents of nitroalkane and 0.1 equivalent of DIPEA (9 μL) were added. The reaction mixture was left stirring for the 24 h, after that the volatile compounds were removed under reduced pressure and the residue was directly purified on silica gel column eluting with hexane:EtOAc to afford the corresponding product.

#### 3.2.1. *R*-(−)-1-Nitro-2-hexanol (**6a**)

Purified by column chromatography on silica (hexane:EtOAc 9:1), colorless oil, 90% yield, 57% ee. Enantiomeric excess was determined by HPLC (Chiralcel OD-H column, hexane/isopropanol, 90/10 *v*/*v*, 0.5 mL/min, 210 nm): t_R (major)_ = 8.34 min, t_R (minor)_ = 9.95 min; ${\left[\alpha \right]}_{D}^{20}$ = −6.5° (c = 1.2, CH_2_Cl_2_), (lit. [21] ${\left[\alpha \right]}_{D}^{20}$ = −9.3° (c = 2.73, CH_2_Cl_2_, 93% ee (*R*))).^1^H-NMR (CDCl_3_, 300 MHz, TMS): δ = 4.44–4.25 (m, 3H), 2.51 (d, *J* = 4.7 Hz, 1H), 1.49–1.31 (m, 6H), 0.90 (t, *J* = 7.0 Hz, 3H). ^13^C-NMR (100 MHz, CDCl_3_, TMS): δ = 80.6, 68.7, 33.4, 27.3, 22.4, 13.9 ppm. 

#### 3.2.2. *R*-(+)-4-Methyl-1-nitropentan-2-ol (**6b**)

Purified by column chromatography (hexane:EtOAc 10:1), colourless oil, 89% yield, 49% ee. Enantiomeric excess was determined by HPLC (Chiralcel AD-H column, hexane/isopropanol, 90/10 *v*/*v*, 0.5 mL/min, 23 °C, 210 nm): t_R (major)_ = 13.53 min, t_R (minor)_ = 17.72 min; ${\left[\alpha \right]}_{D}^{20}$ = +1.0° (c = 1.0, CH_2_Cl_2_), (lit. [4] ${\left[\alpha \right]}_{D}^{20}$ = +2.3° (c = 2.5, CH_2_Cl_2_, 82% ee (*R*))). ^1^H-NMR (CDCl_3_, 400 MHz, TMS): δ = 4.40–4.32 (m, 3H), 2.53 (d, *J* = 4.0 Hz, 1H), 1.84–1.78 (m, 1H), 1.51–1.44 (m, 1H), 1.23–1.17 (m, 1H), 0.94 (app t, *J* = 6.2 Hz, 6H). ^13^C-NMR 100 MHz, CDCl_3_, TMS): δ = 81.0, 67.0, 42.4, 24.3, 23.2, 21.7.

#### 3.2.3. *R*-(−)-3,3-Dimethyl-1-nitrobutan-2-ol (**6c**)

Purified by column chromatography (hexane:EtOAc 10:1), colourless oil, 88% yield, 67% ee. Enantiomeric excess was determined by HPLC (Chiralcel OD-H column, hexane/isopropanol, 98/2 *v*/*v*, 1.0 mL/min, 23 °C, 210 nm): t_R (major)_ = 16.39 min, t_R (minor)_ = 19.31 min; ${\left[\alpha \right]}_{D}^{20}$ = −29.3° (c = 0.15, CH_2_Cl_2_), (lit. [57] ${\left[\alpha \right]}_{D}^{20}$ = −28.1° (c = 1.78, CH_2_Cl_2_, 69% ee (*R*))). ^1^H-NMR (CDCl_3_, 400 MHz, TMS): δ = 4.5 (dd, *J_1_* = 13.0 Hz, J_2_ = 2.0 Hz, 1H), 4.35 (dd, *J_1_* = 13 Hz, J_2_ = 10.1 Hz, 1H), 4.01 (ddd, *J_1_* = 10.1 Hz, *J_2_* = 4.7 Hz, J_3_ = 2 Hz), 2.39 (d, *J* = 4.7 1H), 0.96 (s, 9H). ^13^C-NMR (75 MHz, CDCl_3_, TMS): δ = 78.2, 76.2, 34.3, 25.6.

#### 3.2.4. *R*-(−)-3-Methyl-1-nitrobutan-2-ol (**6d**)

Purified by column chromatography (hexane:EtOAc 10:1), colourless oil, 83% yield, 60% ee. Enantiomeric excess was determined by HPLC (Chiralcel OD-H column, hexane/isopropanol, 98/2 *v*/*v*, 1.0 mL/min, 23 °C, 210 nm): t_R (major)_ = 20.46 min, t_R (minor)_ = 22.90 min; ${\left[\alpha \right]}_{D}^{20}$ = −17.9° (c = 0.2, CH_2_Cl_2_), (lit. [45] ${\left[\alpha \right]}_{D}^{20}$ = −30.6° (c = 0.69, CH_2_Cl_2_, 99% ee (*R*))). ^1^H-NMR (CDCl_3_, 400 MHz, TMS): δ = 4.53–4.36 (m, 2H), 4.10–4.07 (m, 1H), 2.51 (br s, 1H), 1.82–1.74 (m, 1H), 0.97 (app t, *J* = 6.3 Hz). ^13^C-NMR (100 MHz, CDCl_3_, TMS): δ = 79.3, 73.4, 31.7, 18.4, 17.5.

#### 3.2.5. *R*-(−)-1-Nitroundecan-2-ol (**6e**) 

Purified by column chromatography (hexane:EtOAc 10:1), yellowish oil, 82% yield; 55% ee. Enantiomeric excess was determined by HPLC (Chiralcel AD-H column, hexane/isopropanol, 90/10 *v*/*v*, 0.5 mL/min, 23 °C, 210 nm): t_R (major)_ = 13.19 min, t_R (minor)_ = 17.99 min; ${\left[\alpha \right]}_{D}^{20}$ = −2.8° (c = 1.9, CH_2_Cl_2_), (lit. [45] ${\left[\alpha \right]}_{D}^{20}$ = −4.9° (c = 1.04, CH_2_Cl_2_, 97% ee (*R*))). ^1^H-NMR (CDCl_3_, 400 MHz, TMS): δ = 4.43–4.28 (m, 3H), 2.47 (d, *J* = 4.5 Hz, 1H), 1.57–1.44 (m, 3H), 1.37–1.24 (m, 13H), 0.86 (t, *J* = 6.7 Hz, 3H). ^13^C-NMR (100 MHz, CDCl_3_, TMS): δ = 80.6, 68.7, 33.7, 31.9, 29.5, 29.4, 29.3, 29.3, 25.2, 22.7, 14.1.

#### 3.2.6. *R*-(−)-1-Nitrotridecan-2-ol (**6f**)

Purified by column chromarography (hexane:EtOAc 10:1), pale yellow oil, 75% yield, 57% ee. Enantiomeric excess was determined by HPLC (Chiralcel AD-H column, hexane/isopropanol, 90/10 *v*/*v*, 0.5 mL/min, 23 °C, 210 nm): t_R (major)_ = 11.51 min, t_R (minor)_ = 14.79 min; ${\left[\alpha \right]}_{D}^{20}$ = −2.5° (c = 0.4, CH_2_Cl_2_). ^1^H-NMR (CDCl_3_, 400 MHz, TMS): δ = 4.35–4.32 (m, 2H), 4.31–4.27 (m, 1H), 2.47 (d, *J* = 4.6 Hz, 1H), 1.56–1.44 (m, 2H), 1.34–1.24 (m, 18H), 0.86 (t, *J* = 6.7 Hz). ^13^C-NMR (100 MHz, CDCl_3_, TMS): δ = 80.6, 68.7, 33.7, 31.9, 29.6, 29.5, 29.4, 29.3 (2H), 25.2, 22.7, 14.1. 

#### 3.2.7. *R*-(−)-(*E*)-1-Nitroundec-3-en-2-ol (**6g**) 

Purified by column chromatography (hexane:EtOAc 95:5), yellow oil, 65% yield, 47% ee. Enantiomeric excess was determined by HPLC (Chiralcel AD-H column, hexane/isopropanol, 98/2 *v*/*v*, 0.8 mL/min, 23 °C, 210 nm): t_R (major)_ = 28.80 min, t_R (minor)_ = 30.60 min; ${\left[\alpha \right]}_{D}^{20}$ = −0.4° (c = 0.7, CH_2_Cl_2_). ^1^H-NMR (CDCl_3_, 400 MHz, TMS): δ = 5.86 (dtd, *J_1_* = 15.4 Hz, *J_2_* = 6.8 Hz, *J_3_* = 1.1 Hz, 1H), 5.42 (ddt, *J_1_* = 15.4 Hz, *J_2_* = 6.6 Hz, *J_3_* = 1.5 Hz, 1H), 4.79 (pentet, *J* = 5.6 Hz, 1H), 4.40 (d, *J* = 5.7 Hz, 2H), 2.40 (d, *J* = 4.4 Hz, 1H), 2.03 (dd, *J_1_* = 14.2 Hz, *J_2_* = 7.2 Hz, 2H), 1.37–1.25 (m, 10H), 0.86 (t, *J* = 6.7 Hz, 3H). ^13^C-NMR (100 MHz, CDCl_3_, TMS): δ = 136.3, 126.0, 8.1, 70.0, 32.2, 31.8, 29.1, 29.1, 28.8, 22.6, 14.1.

#### 3.2.8. (*R*)-(−)-1-Phenyl-2-nitroethanol (**6h**)

Prepared according general procedure and purified by column chromatography (hexane:EtOAc, 8:1), colorless oil, 63% yield, 45% ee. Enantiomeric excess was determined by HPLC (Chiralcel OD-H column, hexane/isopropanol, 90/10 *v*/*v*, 0.5 mL/min, 254 nm): t_R (major)_ = 19.88 min, t_R (minor)_ = 24.00 min. ${\left[\alpha \right]}_{D}^{20}$ = −23.1° (c = 1.2 CH_2_Cl_2_), (lit. [45] ${\left[\alpha \right]}_{D}^{20}$ = −53.1° (c = 1.06 CH_2_Cl_2_, 96% ee (*R*))). ^1^H-NMR (CDCl_3_, 300MHz, TMS): δ = 7.40–7.34 (m, 5H), 5.46 (dt, *J_1_* = 9.4 Hz, *J_2_* = 3.5 Hz, 1H), 4.60 (dd, *J_1_* = 13.3 Hz, *J_2_* = 9.3 Hz, 1H), 4.50 (dd, *J_1_* = 13.4 Hz, *J_2_* = 3.2 Hz, 1H), 2.78 (br s, 1H). ^13^C-NMR (75 MHz, CDCl_3_, TMS): δ = 138.2, 129.0, 128.9, 126.0, 81.2, 71.0.

#### 3.2.9. (2*R*,3*R*)-5-Methyl-2-nitrohexane-3-ol (**7a**)

Prepared according general procedure and purified by column chromatography (hexane:EtOAc 10:1), colourless oil, 93% yield. Enantiomeric excess was determined by HPLC (Chiralcel AD-H column, hexane/isopropanol, 98/2 *v*/*v*, 1.0 mL/min, 23 °C, 220 nm): t_R_ (*anti* minor) = 16.40 min, t_R_ (*anti* major) = 17.62 min, t_R_ (*syn* major) = 21.28 min, t_R_ (*syn* minor) = 22.85 min. ${\left[\alpha \right]}_{D}^{20}$ = +15.2° (c = 0.91 EtOH), (lit. [58] ${\left[\alpha \right]}_{D}^{20}$ = +12.5° (c = 0.96 EtOH, *syn*/*anti* 93/7, 99% ee)). Diastereomeric ratio (*syn*/*anti*) was determined by ^1^H-NMR (CDCl_3_, 400 MHz, TMS): δ = 4.52–4.43 (m, 1H), 4.25–4.22 (m, 0.42H, *anti*), 3.96–3.91 (m, 0.58H, *syn*), 2.18 (br s, 1H), 1.88–1.76 (m, 1H), 1.53 (dd, *J*_1_ = 6.7 Hz, *J*_2_ = 3.8 Hz, 3H), 1.42–1.35 (m, 1H), 1.24–1.16 (m, 1H), 0.95–0.90 (m, 6H). ^13^C-NMR (100 MHz, CDCl_3_, TMS): δ = 88.1 (*syn*), 86.7 (*anti*), 71.2 (*syn*), 70.2 (*anti*), 42.0 (*syn*), 41.8 (*anti*), 24.6 (*anti*), 24.3 (*syn*), 23.6 (*syn*), 23.3 (*anti*), 21.7 (*anti*), 21.4 (*syn*), 16.2 (*syn*), 12.4 (*anti*).

#### 3.2.10. (3*R*, 4*R*)-2-methyl-4-nitropentan-3-ol (**7b**)

Prepared according general procedure and purified by column chromatography (hexane:EtOAc 10:1), colourless oil, 94% yield. Enantiomeric excess was determined by HPLC (Chiralcel OD-H column, hexane/isopropanol, 99/1 *v*/*v*, 1.0 mL/min, 23 °C, 220 nm): t_R_ (*anti* major) = 18.56 min, t_R_ (*syn* major) = 19.26 min, t_R_ (*anti* minor) = 21.66 min, t_R_ (*anti* minor) = 22.36 min. ${\left[\alpha \right]}_{D}^{20}$ = −3.4° (c = 1.12 CHCl_3_), (lit. [58] ${\left[\alpha \right]}_{D}^{20}$ = −2.0° (c = 0.85 CHCl_3_, *syn*/*anti* 97/3, 90% ee)). Diastereomeric ratio (*syn*/*anti*) was determined by ^1^H-NMR (CDCl_3_, 400 MHz, TMS): δ = 4.68–4.61 (m, 1H), 3.86 (dd, *J*_1_ = 8.0 Hz, *J*_2_ = 3.2 Hz, 0.19H, *anti*), 3.67 (dd, *J*_1_ = 7.2 Hz, *J*_2_ = 4.5 Hz, 0.81H, *syn*), 2.18 (br s 1H), 1.82–1.74 (m, 0.81H, *syn*), 1.72–1.64 (m, 0.19H, *anti*), 1.53 (d, *J* = 6.8 Hz, 3H), 1.02 (d, *J* = 6.8 Hz, 3H), 0.91 (d, *J* = 6.7 Hz, 3H). ^13^C-NMR (100 MHz, CDCl_3_, TMS): δ = 86.2 (*syn*), 84.6 (*anti*), 77.3 (*syn*), 77.2 (*anti*), 30.8 (*anti*), 29.9 (*syn*), 19.8 (*anti*), 18.8 (*syn*), 18.6 (*anti*), 16.4 (*syn*), 15.5 (*syn*), 12.1 (*anti*).

## 4. Conclusions 

In conclusion, a new pinane-based dinitrogen C_1_-symmetric ligand has been synthesized *via* an experimental simple one pot procedure. This ligand was found to be reasonably selective for the copper catalyzed asymmetric Henry reaction between nitromethane and aliphatic aldehydes. Of merit, high yields were observed for this class of aldehydes. The ease of availability of ligand **4** in both enantiomeric forms, together with the simple and scalable procedure for its preparation, is of advantage for the present catalytic system. Currently, this catalyst system is investigated towards the synthesis of vicinal amino-alcohol derived lipids.
